# Gene and miRNA expression signature of Lewis lung carcinoma LLC1 cells in extracellular matrix enriched microenvironment

**DOI:** 10.1186/s12885-016-2825-9

**Published:** 2016-10-11

**Authors:** Vaidotas Stankevicius, Gintautas Vasauskas, Danute Bulotiene, Stase Butkyte, Sonata Jarmalaite, Ricardas Rotomskis, Kestutis Suziedelis

**Affiliations:** 1 National Cancer Institute, Vilnius, Lithuania; 2 Department of Biochemistry and Molecular Biology, Faculty of Natural Sciences, Joint Life Sciences Center, Vilnius University, Vilnius, Lithuania; 3 Vilnius University Institute of Biotechnology, Joint Life Sciences Center, Vilnius University, Vilnius, Lithuania; 4 Human Genome Research Centre, Department Botany & Genetics, Faculty of Natural Sciences, Joint Life Sciences Center, Vilnius University, Vilnius, Lithuania; 5 Biophotonics Group of Laser Research Centre, Vilnius University, Vilnius, Lithuania; 6 Laboratory of Molecular Oncology, National Cancer Institute, Santariskiu 1, Vilnius, LT-08660 Lithuania

**Keywords:** 3D cell culture, ECM, Gene and miRNA expression signature, MAPK signaling pathway, Cell adhesion, Inflammatory response

## Abstract

**Background:**

The extracellular matrix (ECM), one of the key components of tumor microenvironment, has a tremendous impact on cancer development and highly influences tumor cell features. ECM affects vital cellular functions such as cell differentiation, migration, survival and proliferation. Gene and protein expression levels are regulated in cell-ECM interaction dependent manner as well. The rate of unsuccessful clinical trials, based on cell culture research models lacking the ECM microenvironment, indicates the need for alternative models and determines the shift to three-dimensional (3D) laminin rich ECM models, better simulating tissue organization. Recognized advantages of 3D models suggest the development of new anticancer treatment strategies. This is among the most promising directions of 3D cell cultures application. However, detailed analysis at the molecular level of 2D/3D cell cultures and tumors in vivo is still needed to elucidate cellular pathways most promising for the development of targeted therapies. In order to elucidate which biological pathways are altered during microenvironmental shift we have analyzed whole genome mRNA and miRNA expression differences in LLC1 cells cultured in 2D or 3D culture conditions.

**Methods:**

In our study we used DNA microarrays for whole genome analysis of mRNA and miRNA expression differences in LLC1 cells cultivated in 2D or 3D culture conditions. Next, we indicated the most common enriched functional categories using KEGG pathway enrichment analysis. Finally, we validated the microarray data by quantitative PCR in LLC1 cells cultured under 2D or 3D conditions or LLC1 tumors implanted in experimental animals.

**Results:**

Microarray gene expression analysis revealed that 1884 genes and 77 miRNAs were significantly altered in LLC1 cells after 48 h cell growth under 2D and ECM based 3D cell growth conditions. Pathway enrichment results indicated metabolic pathway, MAP kinase, cell adhesion and immune response as the most significantly altered functional categories in LLC1 cells due to the microenvironmental shift from 2D to 3D. Comparison of the expression levels of selected genes and miRNA between LLC1 cells grown in 3D cell culture and LLC1 tumors implanted in the mouse model indicated correspondence between both model systems.

**Conclusions:**

Global gene and miRNA expression analysis in LLC1 cells under ECM microenvironment indicated altered immune response, adhesion and MAP kinase pathways. All these processes are related to tumor development, progression and treatment response, suggesting the most promising directions for the development of targeted therapies using the 3D cell culture models.

**Electronic supplementary material:**

The online version of this article (doi:10.1186/s12885-016-2825-9) contains supplementary material, which is available to authorized users.

## Background

The extracellular matrix (ECM), as one of the key components of tumor microenvironment, has a significant impact on cancer development and highly influences tumor cell features and therefore the response to treatment [1]. ECM contributes not only structural support of growing tumor cells, but also affects other cellular functions such as cell differentiation, migration, survival or proliferation [2–4]. Moreover, gene and protein expression levels are regulated in cell-ECM interaction dependent manner [5, 6]. Not surprisingly, clinical trials based on preclinical two-dimensional (2D) monolayer cell culture models which lack representation of ECM dependent molecular processes occurring in tumors currently have a failure rate of up to 95 %. Cancer cell growth under three-dimensional (3D) culture conditions simulating ECM microenvironment better resembles tumor cell properties in vivo [7]. Thus, investigations using such 3D cell culture models are expected to result in more successful clinical trials.

Vast amount of evidence indicates the superiority of 3D cell cultures compared to 2D models for investigating cancer tumor microenvironment dependent cancer cell properties [8, 9]. Obvious advantages of 3D cell culture models are the cellular-ECM interactions and cell-cell contacts, the formation of active proliferation, quiescent viable cell and necrotic cell zones, as well as the formation of nutritional, oxygen and drug gradients better reflecting cellular organization and the microenvironment in tumor tissue [10]. Nevertheless, the 3D cell cultures do not resemble the full complexity of tumor tissue environment in vivo. Few obvious limitations of 3D cell cultures as a cancer research model are the lack of vasculature, host immune response and other cell-cell interactions that occur between cancer and stromal cells in tumors [11]. Recognized advantages and limitations of 3D cell culture models suggest that the most successful directions of 3D model application include the development of new anticancer treatment strategies. Hence, detailed analysis at the molecular level of 2D/3D cell cultures and tumors in vivo are still needed to unlock the power of 3D cell culture models in translational research.

In order to elucidate which biological pathways are altered during microenvironmental shift, we have analyzed whole genome mRNA and miRNA expression changes in murine Lewis lung cancer LLC1 cells cultured in 2D or laminin rich ECM (lr-ECM) 3D conditions. LLC1 cell line was established from the lung of a C57BL mouse bearing a tumor of primary Lewis lung carcinoma. This cell line is highly tumorigenic and the implanted cells are immunologically compatible with the murine immune system, unlike the widely used human cancer xenograft models. Therefore, it is primarily used as singeneic animal model as well as evaluating the efficacy of chemotherapeutic agents in vivo. The present pathway enrichment results indicated the metabolic pathway, MAP kinase, cell adhesion and immune response as the most significantly altered functional categories in LLC1 cells during the switch from 2D to 3D. Global miRNA expression analysis confirmed the involvement of miRNA in the regulation of ECM dependent properties of cancer cells. Comparison of the expression levels of selected genes and miRNA between LLC1 cells grown 3D cell culture and LLC1 tumors implanted in mice indicated correspondence between both model systems. Global gene and miRNA expression analysis indicates the existence of universal regulation for the metabolic pathway, MAPK, cell adhesion and immune response pathways both in 3D culture and tumor suggesting the most promising directions for translational cancer research using the 3D cell culture models.

## Methods

### Cell culture and maintenance

LLC1 mouse Lewis lung carcinoma cell line was obtained from the ATCC (Rockville, Maryland, USA). Cells were cultured under standard conditions at 37 °C in a humidified atmosphere containing 5 % CO_2_ with DMEM medium (ThermoFisher Scientific, USA) supplemented with 10 % fetal bovine serum (ThermoFisher Scientific, USA), 2 mM glutamine (ThermoFisher Scientific, USA), 100 UI/ml penicillin (Sigma, USA) and 0.1 mg/ml streptomycin (Sigma, USA). For 2D culture, cells were plated in 6 well plates at 5x10^4^ cells/cm^2^ density. For lr-ECM 3D cell culture, 24 well plates were coated with 1 % agarose to prevent the attachment of cells to the plate bottom and 5x10^4^ cells per well were embedded into 0.5 mg/ml lr-ECM protein mixture Geltrex (ThermoFisher Scientific, USA) in DMEM medium as described previously [12]. All experiments were performed following 48 h of cell growth and repeated at least 3 times. Representative phase contrast images of live LLC1 cells grown under 2D and lr-ECM 3D cell culture conditions were taken using Nikon T5100 microscope.

### Tumor model

C57BL/6 female mice (obtained from Vilnius University Institute of Biochemistry) at 10–12 weeks of age and 19–22 g body weight were used. Mice were injected subcutaneously with Lewis lung carcinoma (LLC1) cells (1x10^6^ cells suspended in RPMI medium) in the right groin. Animals were sacrificed, tumors excised, homogenized and resuspended in normal saline 10 days following the implantation. Experimental group of mice were injected with 0.2 ml of the obtained suspension in the right groin. Mice were housed at a constantly maintained temperature (22 ± 1 °C), relative humidity (55 ± 10 %) and photoperiod (12 h light/dark cycle) in the Open Access Centre at National Cancer Institute, Lithuania. The animals were fed standard rodent chow and purified water ad libitum. Tumor volume was determined by measuring the diameter with vernier calipers and calculating the volume according to the following formula: tumor volume = L x W x H x π/6 (L is length, W is width and H is height of tumor). Tumors reached 400–600 mm^3^ volume in 10 days following implantation. Then animals were sacrificed and tumors excised and used for total RNA isolation. All animal procedures were performed in accordance with the guidelines established by the Lithuanian Care Committee which approved the study (No.0190)

### Confocal imaging

5x10^4^ LLC1 cells were plated in 24 well plates on glass cover slips in DMEM or embedded into 0.5 mg/ml lr-ECM/DMEM mixture under 2D or 3D cell culture conditions, respectively. Following 48 h of growth, cells were washed twice with PBS and fixed for 10 min. with 4 % PFA (Carl ROTH, Germany) solution in PBS at room temperature. Cell permeabilization was performed with ice-cold 0,1 % Triton X-100 in PBS for 10 min. Staining was accomplished with Alexa®633 Phalloidin (ThermoFisher Scientific, USA) in PBS containing 1 % BSA for 30 min and 5 μg/ml Dapi (Sigma, USA) in PBS for 3 min at room temperature. All staining steps were followed by 3 wash steps in PBS for 5 min at room temperature. Finally, slides were mounted with Roti®-MountFluorCare mounting media (Carl ROTH, Germany). Images were obtained using Zeiss LSM 7 Duo Live confocal microscope (Zeiss, Germany) and 40x/1.3 immersion objective and excitation wavelengths of 405 nm and 633 nm.

### RNA and miRNA extraction

1x10^6^ LLC1 cells following 48 h of growth under 2D or lr-ECM 3D cell culture conditions were harvested and total RNA enriched with small noncoding RNAs was isolated using mirVana RNA isolation kit (Ambion, USA) according to manufacturer’s instructions. 100 mg of mouse tumor tissue sample were used for total RNA extraction. The quantity and quality of RNA were measured using Nanodrop (ThermoFisher Scientific, USA) and Bioanalyzer (Agilent Technologies, USA).

### Gene expression microarrays

cRNA sample preparation, labeling and hybridization was performed according to manufacturer’s instructions. Briefly, 1 μg of total RNA was used for cDNA synthesis and amplification using Message™Amp aRNA kit (ThermoFisher Scientific, USA). Then 825 ng of cRNA labeled with Cy3/Cy5 dyes using Arcturus® TURBO labeling™ Cy™3/Cy™5 Kit (ThermoFisher Scientific, USA) were hybridized to Agilent Mouse Whole Genome 4x44k Oligonucleotide Microarrays (Agilent Technologies, USA) using HS 400 hybridization station (Tecan, Switzerland). Three independent replicates of every sample were used. Microarray slides were scanned using LS Reloaded scanner (Tecan, Switzerland). Microarray image analysis and data generated were further analyzed using ImaGene ver. 9.0 (BioDiscovery, USA) and GeneSpring GX v11.5 software (Agilent Technologies, USA). Raw extracted gene expression data were normalized with Loess normalization to adjust microarray data for variation. Genes that showed expression values above fold change 1.5 (with *p*-value <0.05) were defined as differentially expressed in LLC1 cells between 2D and 3D cell culture conditions. Microarray design and data are available at the GEO database (Accession No. GSE75863 http://www.ncbi.nlm.nih.gov/geo/query/acc.cgi?acc=GSE75863).

### MiRNA expression microarrays

miRNA labeling was performed using miRNA complete labeling and Hyb kit (Agilent technologies, USA) according to manufacturer’s instructions. In brief, 100 ng of total RNA were dephosphorylated and directly labeled with Cy3. Samples were dried out and resuspended in Hi-RPM hybridization buffer (Agilent Technologies, USA), containing GE blocking agent (Agilent Technologies, USA) and denaturized by heating for 5 min in 100 °C. In a further step samples were hybridized to Agilent mouse miRNA 8x15K microarrays containing probes for 627 mouse miRNAS from Sanger database v.12 (Agilent Technologies, USA) for 20 h at 55 °C in a rotating hybridization oven. Three independent replicates of every sample were used. Slides were then washed 3 times and scanned with Agilent SureScan Microarray Scanner (Agilent Technologies, USA). Microarray images were extracted using Extraction Feature v10.7.3.1 software (Agilent Technologies, USA). To normalize raw probe values, experimental samples were scaled to mean of control samples using GeneSpring GX v11.5 software (Agilent Technologies, USA). miRNAs that showed expression values above fold change 2 (with *P*-value <0.05) were defined as differentially expressed in LLC1 cells between 2D and 3D cell culture conditions. Microarray data are available at the GEO database (Accession No. GSE75862, http://www.ncbi.nlm.nih.gov/geo/query/acc.cgi?acc=GSE75862).

### Microarray data enrichment analysis

To classify microarray data we selected KEGG pathway enrichment analysis which provided the most extensive pathway enrichment results compared to other open access toolkits. KEGG pathway enrichment analysis of gene expression data was performed using WebGestalt online source, as described previously [13]. *P* values were calculated using hypergeometric test and adjusted with multiple Benjamini and Hochberg testing. Functional categories associated in at least 5 genes and *P* < 0.05 were defined as significant. miRNA target and KEGG pathway enrichment analysis was performed with Diana Tools using microT-CDS algorithm and MirPath, as described previously [14, 15].

### Quantitative RT-PCR

To validate gene expression changes, 1 μg of total RNA was used for cDNA synthesis with RevertAid RT Kit (ThermoFisher Scientific, USA) according to manufacturer’s instructions. To evaluate miRNA expression, 0.2 μg of total RNA was used to perform cDNA synthesis with RevertAid RT Kit (ThermoFisher Scientific, USA) as described previously [16]. Quantitative real-time PCR (qRT-PCR) was performed on Eco™ RT-PCR system (Illumina, Inc.) using 2x Maxima SYBR Green qPCR MasterMix (ThermoFisher Scientific, USA) according to manufacturer’s instructions. The relative changes in gene and miRNA expression were calculated by ∆∆C_t_ method comparing expression levels in LLC1 cells grown under 2D and lr-ECM 3D or LLC1 tumors with *hprt1* or sno135 as endogenous controls for expression normalization, respectively. The primer sequences used for microarray data validation are shown in Additional file 1: Table S1.

### Statistical analysis

Data were analyzed using GraphPad v6.0 software. Student’s *t* test was used to compare differences between two groups. *P* < 0.05 was considered as statistically significant. All experiments were independently repeated at least 3 times.

## Results

### Cell morphology

**Fig. 1:**
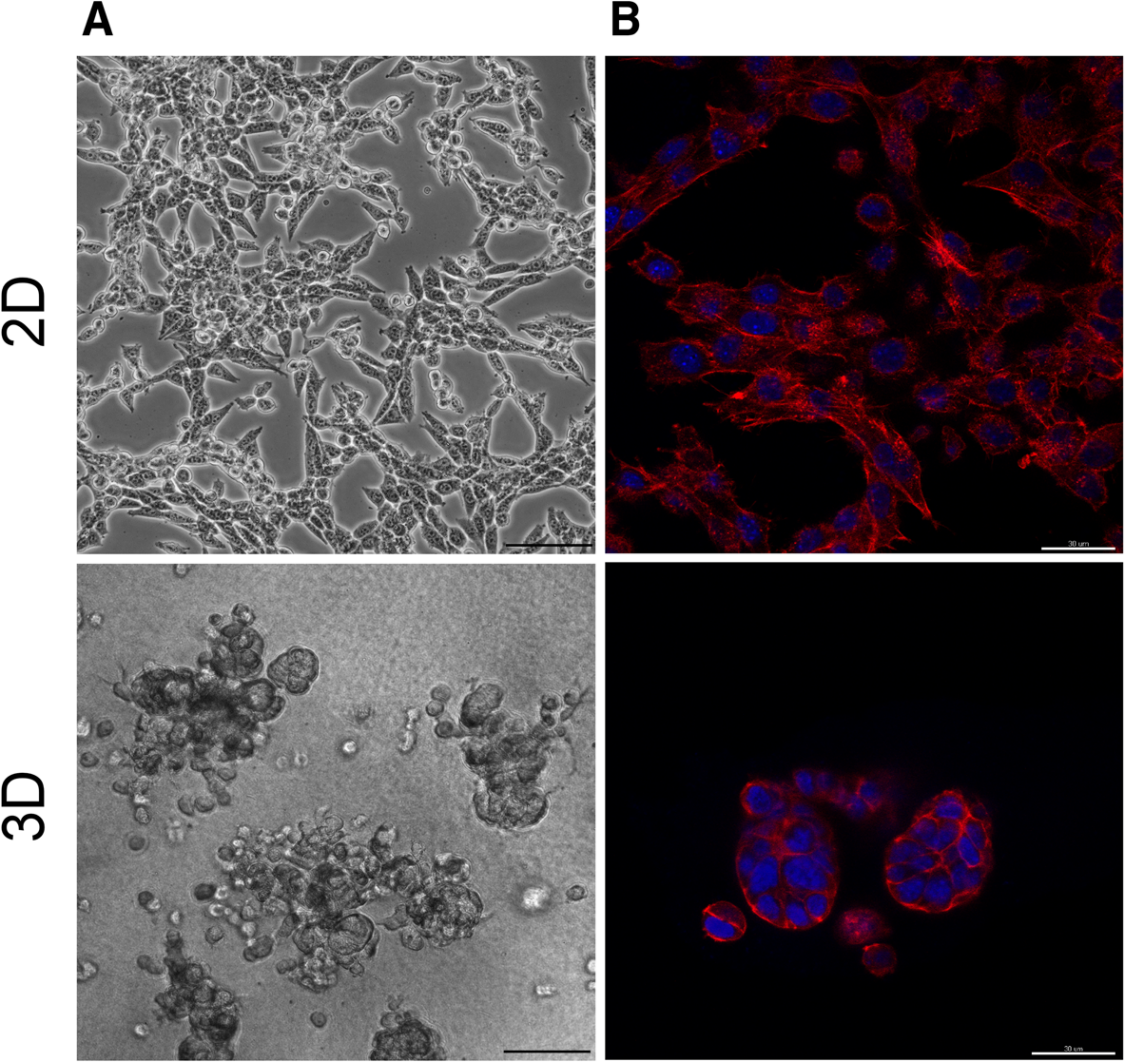
Cell morphology differences of LLC1 cells grown under 2D or lr-ECM 3D growth conditions. Prior to imaging cells were grown under 2D (*upper* panel) and lr-ECM 3D (*lower* panel) cell culture conditions for 48 h. Representative phase contrast (**a**) and confocal laser scanning microscopy images (**b**) of cells under 2D and 3D growing conditions. F-actin was stained with AlexaFluor 633 Phaloidin (*red*). Nuclei were counterstained with DAPI (*blue*). Bars 100 μm (*upper* panel) and 30 μm (*lower* panel)

To elucidate structural changes in cellular morphology we examined mouse Lewis lung carcinoma LLC1 cells grown under 2D or lr-ECM 3D cell culture conditions. Cells lost their flat elongated morphology and gained 3-dimensional characteristic mass view following 48 h of cell growth in 3D conditions (Fig. 1a). Further, in order to visualize cell actin cytoskeleton changes, we also performed confocal microscopy of cells stained with phalloidin (Fig. 1b). Images showed that cells had undergone a significant actin cytoskeleton rearrangement and actin stress fibers were lost under 3D culture conditions.

### Gene expression pattern in LLC1 cells grown under lr-ECM 3D conditions

To better understand the impact of cellular microenvironment changes on gene expression levels in LLC1 cells grown under 2D and lr-ECM 3D conditions, we analyzed genome wide expression changes between these culture conditions using Agilent Mouse Whole Genome 4x44k Oligonucleotide Microarray platform. Microarray data revealed that the expression of 1884 genes was significantly altered (>1.5 fold change, *p* < 0.05) following 48 h cell growth under 2D and lr-ECM 3D conditions (Additional file 2: Table S2). Differences in cell culture conditions resulted in a greater number of down-regulated than up-regulated genes (1052 and 832, respectively; Table 1). In order to evaluate which biological pathways were affected in LLC1 cells between 2D and 3D cell growth conditions, we performed KEGG pathway enrichment analysis of all 1884 differently expressed genes. KEGG pathway enrichment analysis revealed that total of 74 KEGG pathway categories were enriched where each of these categories was represented at least by five or more genes in the functional category with *p* < 0.05 (Additional file 3: Table S3). A greater number of genes in all categories were down-regulated in LLC1 cells under 3D culture conditions as compared to 2D. Next, we coalesced similar KEGG pathway categories into four subsets of major functional groups, which could be related to tumor development and progression: 1) Metabolic pathways; 2) MAP kinase; 3) Cell adhesion and 4) Immune response related pathways (Table 2). Our results indicated that the “Metabolic pathways” subset was the most significantly altered KEGG functional category (*p* = 6.23e-08) resulting in 73 (30 up-regulated and 43 down-regulated) differently expressed genes in LLC1 cells grown under lr-ECM 3D vs monolayer conditions. The MAP kinase signaling pathway was the second most significantly altered KEGG categories (*p* = 6.23e-08) and resulted in 25 genes (11 up-regulated and 14 down-regulated) differently expressed in LLC1 cells cultured under lr-ECM 3D and 2D culture conditions. Differences in cell culture conditions also altered the expression of 48 unique (22 up-regulated and 26 down-regulated) genes related to cell adhesion. These genes were significantly associated with cell adhesion molecules, gap and tight junctions, ECM-receptor interaction functional categories, with “regulation of actin cytoskeleton” (*p* = 1.35e-06) and “focal adhesion” (*p* = 3.33e-05) categories being the most significantly altered in the “cell adhesion” subset. Furthermore, we indicated that difference in cell culture conditions also altered the expression of 44 (16 up-regulated and 28 down-regulated) genes involved in immune response signaling pathways including cytokine-cytokine receptor, chemokine, T and B cell receptor and Jak-STAT signaling pathway categories (Table 2). Differently expressed genes associated to MAP kinase, cell adhesion and immune response pathway categories are listed in Additional file 4: Table S4.

**Table 1:** Number of differentially expressed genes and miRNAs^a^ in LLC1 cells after 48 h growth under 2D and lr-ECM 3D conditions

	All	Up-regulated	Down-regulated
Genes	1884	832	1052
miRNAs	77	41	36

^a^Gene and miRNA expression values are above 1.5 and 2 fold change, respectively, *p* < 0.05

**Table 2:** KEGG pathway enrichment analysis of genes differently expressed in LLC1 cells between 2D and lr-ECM 3D cell culture conditions

Category groups	All		Up-regulated		Down-regulated	
	Genes	*p* value	Genes	*p* value	Genes	*p* value
Metabolic pathways						
Metabolic pathways	73	2.83e–13	30	7.55e–06	43	2.02e–08
MAP Kinase						
MAPK signaling pathway	25	6.23e–08	11	0.0010	14	0.0002
Cell adhesion						
Regulation of actin cytoskeleton	20	1.35e–06	6	0.0404	14	3.76e–05
Focal adhesion	17	3.33e–05	7	0.0148	10	0.0018
Cell adhesion molecules (CAMs)	10	0.0062	5	0.0368	5	NS
Gap junction	7	0.0103	2	NS	5	0.0174
Tight junction	9	0.0103	5	NS	4	NS
ECM-receptor interaction	6	0.0237	4	0.0257	2	NS
Immune response						
Cytokine-cytokine receptor interaction	18	0.0001	6	NS	12	0.0011
T cell receptor signaling pathway	11	0.0003	3	NS	8	0.0011
VEGF signaling pathway	9	0.0004	5	0.0062	4	NS
Cytosolic DNA–sensing pathway	7	0.0013	4	0.0111	3	NS
B cell receptor signaling pathway	7	0.0058	3	NS	4	NS
RIG-I-like receptor signaling pathway	6	0.0121	5	0.0045	1	NS
Natural killer cell mediated cytotoxicity	8	0.0153	3	NS	5	0.0435
Jak–STAT signaling pathway	9	0.0159	3	NS	6	0.0336
Fc epsilon RI signaling pathway	6	0.0186	2	NS	4	NS
Chemokine signaling pathway	9	0.0362	3	NS	6	NS
Toll-like receptor signaling pathway	5	0.0401	4	NS	2	NS

Functional groups of all genes, differentially expressed in LLC cells grown under 3D cell culture conditions, were assign as significant when enriched in at least 5 genes, *p* < 0.05

### miRNA expression pattern in LLC1 cells grown under lr-ECM 3D conditions

**Fig. 2:**
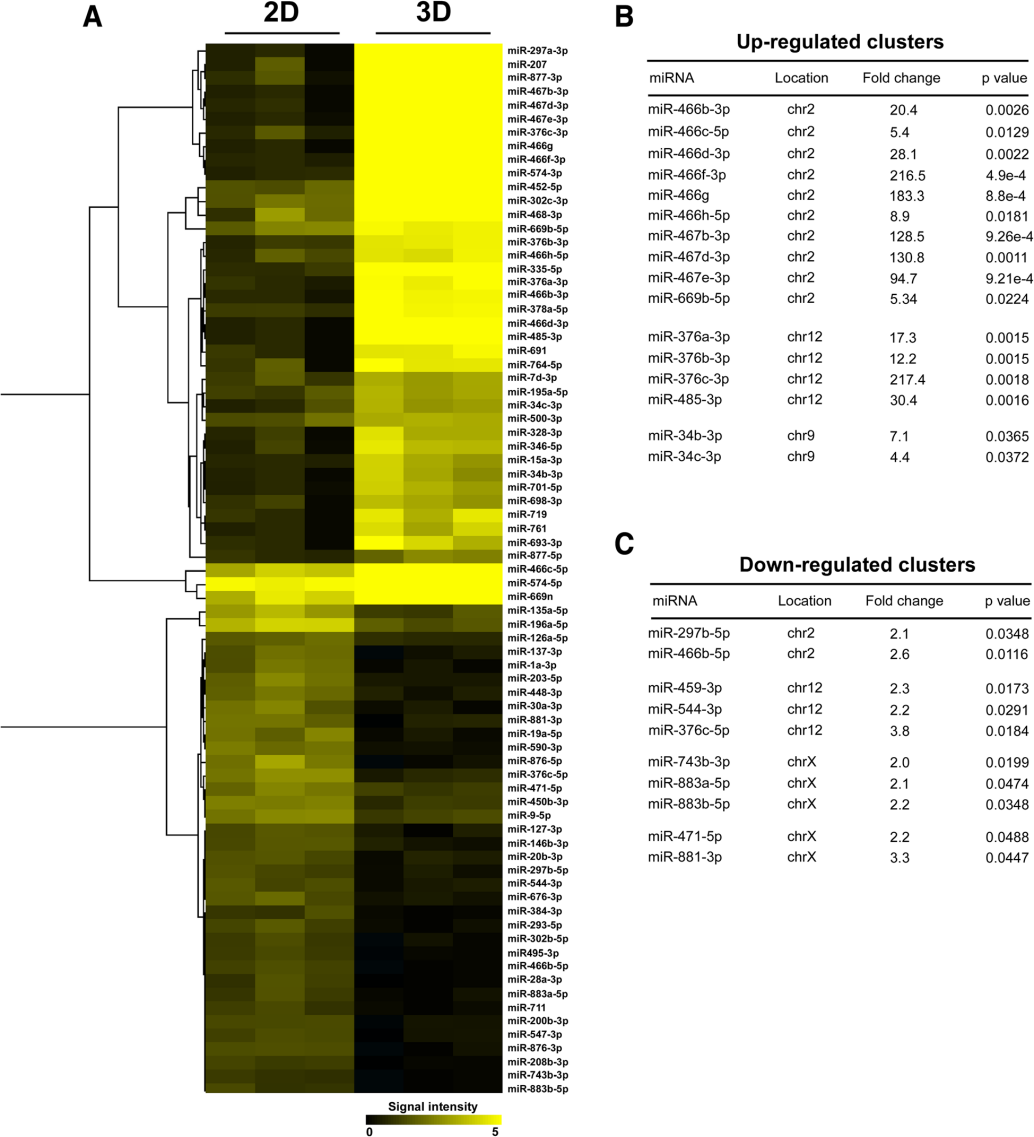
miRNAs regulated in LLC1 cells grown under 2D and lr-ECM 3D cell culture conditions. **a**) Hierarchical clustering depicting differently expressed miRNas (>2 fold change, *p* < 0.05) in LLC1 cells grown under 2D and 3D cell culture conditions. **b**) List of up-regulated miRNA clusters and **c**) down-regulated miRNA clusters in LLC cells grown under lr-ECM 3D cell culture conditions as compared to 2D

For miRNA expression profile analysis, we evaluated miRNA expression changes under two different cell culture conditions using Mouse miRNA 8x15k Microarrays. Following 48 h of cell growth the expression of 77 miRNAs was significantly altered (>2 fold change, *p* < 0.05) and resulted in 41 up-regulated and 36 down-regulated miRNAs in LLC1 cells cultivated under lr-ECM 3D culture conditions compared to miRNA expression levels in cells cultured on plastic (Table 1, Additional file 5: Table S5). Next, to obtain a better overview of miRNA expression signature, we further performed unsupervised hierarchical clustering heat map analysis of all differentially expressed miRNA by normalized probe signal values (Fig. 2a). Heat map analysis revealed: a) the expression of 27 miRNAs was strongly induced under lr-ECM 3D culture conditions and only 3 of these miRNAs, miR-466c, miR-574 and miR-669n showed high expression values under 2D cell culture conditions; b) expression of most miRNAs that were down-regulated under lr-ECM 3D culture conditions showed moderate to low expression values in cells grown in 2D monolayer, except expression of miR-135a and miR-196a. We next checked which members of miRNA cluster were co-expressed. We found that 16 up-regulated miRNAs were associated to 3 clusters, located in chromosome 2 (miR-466 ~ 467 ~ 669 cluster), 9 (miR-34cluster) and 12 (miR-376 cluster) (Fig. 2b) while members (10 miRNAs) of 3 miRNA clusters located in chromosomes 2, 12 and X were down-regulated (Fig. 2c). Interestingly, about 30 % of down-regulated miRNAs were located in chromosome X.

### RNA-miRNA regulatory network analysis

To better understand the biological processes which could be regulated by 77 miRNAs deregulated in LLC1 cells between 2D and 3D cell culture conditions, we indentified 8629 unique target genes potentially regulated by these miRNA using *in silico* miRNA target analysis (Additional file 6: Table S6). Next, miRNA pathway enrichment analysis indicated 69 KEGG categories significantly enriched in targeted genes revealing that pathways related to MAPK, cell adhesion and immune response were also among the most significantly altered functional categories (Additional file 7: Table S7). Furthermore, hierarchical clustering analysis of differently expressed miRNA-associated KEGG pathways also revealed that some miRNAs displayed a similar pathway regulation pattern (Additional file 8). For example, most up-regulated miRNAs of mir-466 ~ 467 ~ 669 cluster were functionally associated and miR-467b/miR-467d/miR-467e, miR-297a/miR-466d showed almost identical patterns. However, hierarchical clustering analysis didn’t indicate any clear correlations of pathway patterns of down-regulated miRNAs (Additional file 8).

Finally, we investigated correlations between differently expressed genes and miRNAs related to Metabolic pathways, MAP kinase, Cell adhesion and Immune response subsets which were the most significantly altered in ECM dependent manner to indicate any potential miRNA-mRNA connections in these processes (Table 3). Our results identified a negative correlation between differential expression of 17 miRNAs and 16 mRNAs from the metabolic pathway category. In the MAP kinase pathway a negative correlation was observed between differential expression of 11 miRNAs and 7 mRNAs. In addition, 14 mRNA targets associated with cell adhesion pathways reversely correlated with 18 miRNAs. Target analysis also revealed that 6 differentially expressed genes from the immune response category reversely correlated with 13 miRNAs.

**Table 3:** Target genes and miRNAs from Metabolic pathways, MAP kinase, Cell Adhesion and Immune Response category groups showing inverse correlation in LLC1 cells after 48 h growth between 2D and lr-ECM 3D cell culture conditions

Category	Up-regulated genes	Down-regulated miRNAs	Down-regulated genes	Up-regulated miRNAs
Metabolic pathways	**Agpat3↑** **Cyp4f18↑**	miR-19a-5p↓	B3gat1↓	**miR-669b-5p↑**
	**Akr1b7↑**	miR-137-3p↓	Dhrs9↓	**miR-297a-3p↑**; **miR-466b-3p↑**; **miR-466d-3p↑**
	**B3galt6↑** **Dtymk↑**	miR-495-3p↓	Kynu↓	**miR-672-5p↑**
	**Ctps↑**	miR-544-3p↓	Ocrl↓	**miR-466 g↑**
	**Dgkb↑**	miR-9-5p↓; miR-590-3p↓; miR-126-5p↓	Pla2g2c↓	**miR-468-3p↑**
	**Ext1↑**	miR-19a-5p↓; miR-590-3p↓; miR-9-5p↓; miR-135a-5p↓	Ppt1↓	**miR-346-5p↑**
	**Man2a1↑**	miR-135a-5p↓; miR-495-3p↓	Sc5d↓	**miR-669b-5p↑**
	**Pigm↑**	miR-9-5p↓; miR-495-3p↓; miR-590-3p↓		
MAPK kinase	**Cacna1d ↑**	miR-137-3p↓; miR-448-3p↓; miR-495-3p↓	Kras↓	**miR-761↑**
	**Ikbkg ↑**	miR-137-3p↓	Mknk1↓	**miR-195-5p↑**
	**Traf6↑**	miR-590-3p↓	Pak2↓	**miR-297a-3p↑**
			Sos2↓	**miR-34b-3p↑**; **miR-34c-3p↑**; **miR-466f-3p↑**; **miR-500-3p↑**
Cell adhesion	**Arhgef4↑**	miR-135a-5p↓; miR-448-3p↓; miR-200b-3p↓; miR-20b-3p↓	Tmsb4x↓	**miR-448↑**
	**Col1a1↑**	miR-135a-5p↓; miR-137↓; miR-590↓	Flna↓	**miR-328-3p**; **miR-761↑**
	**Itpr1↑**	miR-544-3p↓	Gnas↓	**miR-877-3p↑**
	**Pard3↑**	miR-495-3p↓	Htr2c↓	**miR-466d-3p↑**
	**Slc9a1↑**	miR-9-5p↓	Pak2↓	**miR-297a-3p↑**
	**Vegfa ↑**	miR-1a↓	Pak3↓	**miR-297a-3p↑**
			Rhoa↓	**miR-466f-3p↑**
			Ssh1↓	**miR-467b↑**
Immune resopnse	**Pard3↑**	miR-495-3p↓	Eif2ak1↓	**miR-500-3p↑**
			Oas3↓	**miR-297a-3p↑**; **miR-466b-3p↑**; **miR-466d-3p↑**; **miR-466f-3p↑**; **miR-466 g↑**; **miR-467d-3p↑**; **miR-467e-3p↑**
			Ppp2r1b↓	**miR-195-5p↑**; **miR-672-5p↑**
			Ppp2r2b↓	**miR-466 g↑**
			Xcr1↓	**miR-669b↑**

Up-regulated genes and miRNAs are shown in bold

### Microarray gene expression data validation

**Fig. 3:**
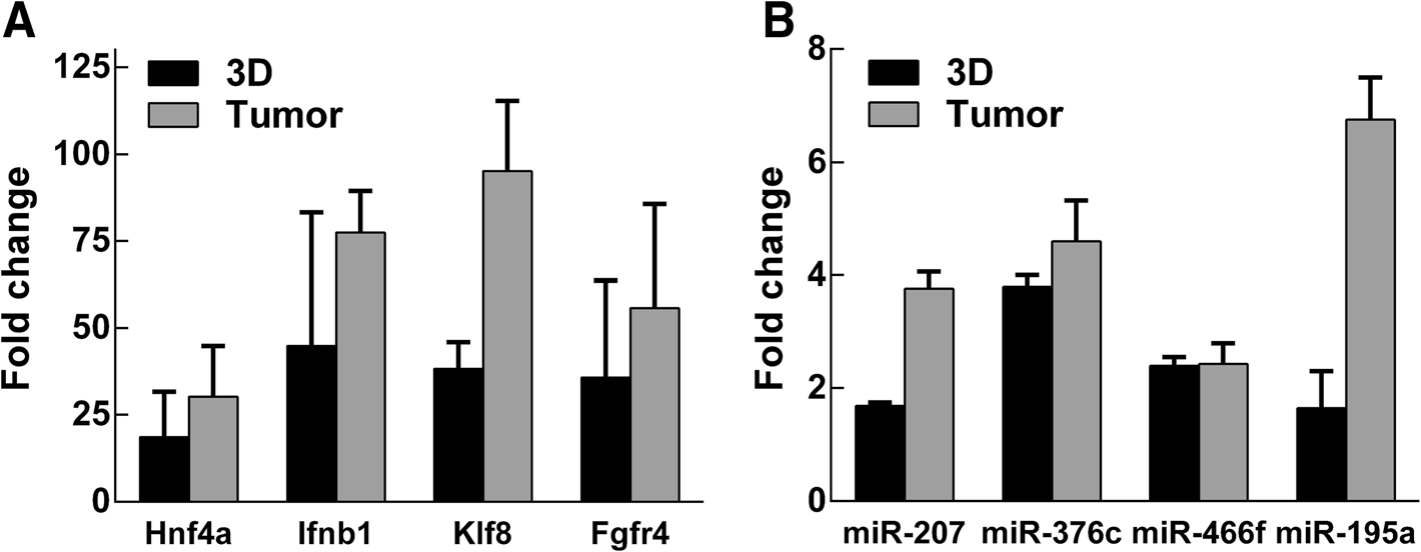
Validation of Microarray gene and miRNA expression data by qPCR. qPCR was performed as described in Methods. qPCR data analysis was based on 2^-ΔΔCt^ method and *gpdh* or sno135 were used as housekeeping genes for gene or miRNA qPCR data normalization, respectively. Graph showing fold changes of **a**) genes (*hnf4a, infb1, klf8* and *fgfr4*) or **b**) miRNAs (miR-207, miR-376c-3p, miR-466f-3p and miR-195a-5p) in LLC1 cells grown under lr-ECM 3D cell culture conditions or in mouse LLC1 tumors compared to expression levels in cells cultivated in 2D. Results show mean ± SD (*n* = 3)

To validate differential expression of genes and miRNAs identified by microarrays, we selected 4 up-regulated genes and miRNAs for qRT-PCR analysis (Fig. 3a and b; black columns). The expression of selected *hnf4a* (Hepatocyte nuclear factor 4a), *ifb1* (Interferon beta-1), *klf8* (Kruppel-like factor 8) and *fgfr4* (Fibroblast growth factor receptor 4) genes was significantly up-regulated in LLC1 cells grown under lr-ECM 3D culture conditions compared to expression levels in cells cultured on plastic. All selected miRNAs, miR-207, miR-376c, miR-466f and miR-195a, also showed significant up-regulation by qPCR. Hence, qRT-PCR data confirmed gene and miRNA microarray data.

Additionally, we also compared the expression of selected genes and miRNAs between 2D monolayer and LLC1 tumors (Fig. 3a and b; grey columns). qRT-PCR analysis clearly showed that all selected genes and miRNAs likewise observed in 3D cell culture conditions were also significantly up-regulated in vivo.

## Discussion

The present study revealed that distinct cellular morphology correlated with an altered gene and miRNA expression profile in mouse Lewis lung carcinoma LLC1 cells grown under lr-ECM 3D cell culture conditions as compared to 2D monolayer. Our results indicated that the ECM strongly affected the expression of particular genes associated with common biological pathways involved in cancer cell adaptation to 3D cell culture microenvironment and correlated with deregulated expression of miRNAs under these conditions. Furthermore, the present study also demonstrated that ECM-enriched cellular microenvironment induced a shift in gene and miRNA expression representative to expression levels *in vivo*. Hence, these results support the application of 3D cell culture to obtain more relevant results for the study of specific miRNAs involved in cell–ECM interaction and of ECM-mediated signaling networks in cancer.

Our findings demonstrated markedly altered gene expression signature of LLC1 cells grown under 2D and lr-ECM 3D cell culture conditions, as it was observed previously in other cell lines [17, 18]. In the present study differences in cell culture conditions resulted in 1884 differently expressed genes demonstrating the broad influence of ECM environment in gene expression regulation. In addition, we also found that the expression of selected *hnf4a*, *infb1*, *klf8* and *fgfr4* genes was significantly increased in LLC1 tumors likewise in LLC1 cells cultured under 3D cell culture conditions compared to gene expression levels in cells grown on plastic. Furthermore, we observed that metabolic, MAP kinase, cell adhesion and immune response functional pathway categories were most significantly altered in LLC1 cells between 2D and 3D culture conditions. The microarray data analysis identified differential expression of 73 genes related to metabolic pathways in LLC1 cells grown under lr-ECM 2D and 3D conditions. We found that the expression of genes involved in pyrimidine/purine, glycerophospholipid, unsaturated fatty acid, amino acid, monosaccharide and drug metabolism were markedly altered in an ECM dependent manner. This indicates that culturing LLC1 cells in 3D cell culture rearranges metabolic functions. In addition, changes in cellular metabolism are tightly connected to pH, nutrient and oxygen gradients leading to the formation of proliferation and hypoxia zones within the tumor microenvironment and 3D cell culture as well [19, 20]. However, genome-wide analyses of metabolic pathway rearrangement in cancer cells grown in an ECM 3D cell culture are limited. Our findings are supported by a previous report that indicated differential expression of genes involved in xenobiotic and lipid metabolism in HepG2 hepatoma cell spheroids suggesting that cells in a 3D culture could be more metabolically active compared to cells grown in monolayer [21]. In addition, Srisomsap et al. [22] revealed signatures of differentially expressed proteins associated with anaerobic glycolysis, mitochondrial and nucleotide metabolism in HepG2 cells grown in a collagen based 3D cell culture. Therefore, altogether these findings suggest that lr-ECM 3D cell culture significantly rearranges metabolic functions in LLC1 cells. Our findings are also in agreement with a previous report also indicating that cellular adaptation to a 3D culture environment significantly alters the expression of genes involved in ECM and cell adhesion [5, 23]. In addition, Luca et al. also observed significantly altered expression of genes involved in MAP kinase pathway [6]. Strikingly, the study also demonstrated altered EGFR protein levels and a switch between RAS-MAPK pathway activation between 2D and lr-ECM 3D environments implying that cellular behavior in different microenvironment could promote important mechanisms to acquire resistance during anticancer therapy. Hence, these findings suggest that the ECM strongly influences the expression of particular genes associated with common biological processes that are involved in cellular adaptation to 3D cell culture conditions. Therefore, results obtained in cells grown under 3D cell culture conditions might also be exploited for the development of targeted cancer therapy.

Furthermore, in our present data we also observed a strong modulation of inflammatory genes in LLC1 cells between 2D and 3D culture conditions. Our findings indicated an altered expression of 44 immune response related genes suggesting that ECM plays an important role in modulating tumor-immune system interactions. Surprisingly, interferon 1 beta (*infb1*) was the most significantly up-regulated inflammatory gene in LLC1 cells under 3D conditions. Interferons have been shown to promote anti-proliferative, anti-angiogenic and immunoregulatory effects on many tumor types [24, 25]. Nevertheless, we also observed increased *ifnb1* levels in mouse LLC1 tumors suggesting that the primary primal role of elevated basal *ifnb1* levels could be more associated with regulation of tumor immuno-surveillance, but not necessarily with tumor suppression. Our results also indicated increased expression of NFAT family *nfatc2* and *nfatc4* genes in LLC1 cells grown under lr-ECM 3D culture conditions as compared to 2D. As NFAT transcription factor family was originally identified to mediate the response of immune cells, recent studies have demonstrated that NFATs also perform important roles in formation of tumor microenvironment. Activation of NFAT signaling in cancer cells results in inflammatory chemokine production eventually leading to recruitment of inflammatory cells to the tumor [26]. Interestingly, recent report suggested that NFAT2 constitutive activation in transgenic mice also linked the microenvironment and the neighboring cells, as both tumor cells expressing NFAT2 and neighboring wild-type cells up-regulated c-Myc and STAT3 in spontaneous skin and ovary tumors [27]. On the other hand, previous reports also associated NFAT signaling axis to *VEGF* driven tumor angiogenesis regulation indicating complex nature of NFAT in metastatic niche formation [28]. In addition, our results also depicted the differential expression of cytokine receptors (*il2ra, il12rb2, il21r* and *il22ra*), chemokine receptors (*ccr3, xrc1* and *cxcr7*) and tumor necrosis factor receptors (*tnfrsf1b, 9, 11a* and *25*) supporting further modulation of cross-talk between cancer and their microenvironment in ECM dependent manner, which cannot be established in 2D cultures. These observations suggest that the investigation of the role of inflammatory genes under 3D cell culture conditions could be very important to understanding the basal influence of genes involved in tumor microenvironment – immune system interactions in vivo. Results obtained culturing cells under 3D cell conditions could be also strongly considered in preclinical targeted therapy research, since ECM environment could strongly influence the responsiveness of tumor cells to immunotherapy.

While it has been well observed that miRNAs regulate the expression of ECM molecules, emerging evidence shows that miRNA expression and function could be significantly affected by the ECM [29, 30]. Consistent with these observations, in the present study microarray data demonstrated a signature of significantly altered expression of 77 miRNAs in LLC1 cells grown under 2D and lr-ECM 3D cell culture conditions compared to cells cultured on plastic. Interestingly, our results showed that ECM strongly induced the up-regulation of miRNA in LLC1 cells grown under 3D culture conditions. This is in accordance with a previous report which suggested that global upregulation of miRNA expression may be linked with the changes in cellular density [31]. Furthermore, our results also indicated that the ECM induced upregulation of miR-466 ~ 467 ~ 669 (e.g. miR-466b,c,d), miR-376 (miR-376a, miR-376b, miR-376c), and miR-34 (miR-34b and miR-34c) clusters. The miR-466 ~ 467 ~ 669 cluster is known as one of the largest miRNA clusters in mouse genome containing 71 miRNAs. A previous report [32] suggested that members of this cluster are abundantly expressed during mouse embryo development and might regulate growth and survival of embryonic stem cells. On the other hand, it has been shown that miR-376 cluster miRNAs are associated with tumorigenesis. For example, elevated expression of miR-376a promoted tumor cell migration and invasion and also positively correlated with advanced tumor metastasis and shorter patient survival [33, 34]. In addition, overexpression of miR-376c increased ovarian cancer cell survival and was associated with poor response to chemotherapy [35]. Moreover, elevated levels of miR-376c were shown in plasma of early stage breast cancer patients [36]. By contrast, miR-34 cluster encodes miRNAs possessing tumor suppressive properties mediating apoptosis, cell cycle arrest and senescence [37]. Our miRNA microarray data were consistent with previous reports indicating that human cancer cells cultured on ECM 3D cell culture conditions have also exhibited a significantly altered miRNA expression profile compared to cells cultured on plastic [38–40]. ECM 3D cell culture associated miRNA profiles demonstrated altered expression of tumor suppressive and oncogenic miRNAs and also correlated with distinct cellular morphogenesis under 3D culture conditions highlighting the regulation of miRNA expression in the ECM dependent manner. Additionally, we also showed that the expression of selected miR-195a, miR-207, miR-376c and miR-466f miRNAs was also significantly increased in mouse LLC1 tumors as compared to miRNA expression levels in 2D indicating the potential role of these miRNAs in tumor progression in vivo. Altogether, these findings suggest that the 3D cell culture should be considered as a critical experimental approach for essential understanding of the miRNA biology associated with tumor microenvironment. Indeed, the gene expression signature of 3D culture of breast cancer cells has been found to define prognostic value for patients with breast cancer [41]. Understanding how ECM regulates miRNA expression will also further elucidate how miRNAs determine tumor development and reveal potential prognostic and therapeutic opportunities.

Further on we also investigated potential relations between 77 differently expressed miRNAs and their target genes to depict possible miRNA-mRNA interactions in LLC1 cells regulated by ECM microenvironment under 3D cell culture conditions. We found that 8629 unique target genes could be regulated by these differently expressed miRNAs. Pathway enrichment analysis also revealed that 69 KEGG pathways were enriched in target genes related to these miRNAs including pathways involved in tumor development. However, as it is known that miRNA targets multiple mRNAs, the ability to find the key pathways by computational approaches is highly dependent on size of miRNA profile. In addition, the statistical target analysis approach could be successful if the miRNA of interest has an effect on the abundance of expressed target gene, but not if expression of target gene is regulated only by translational inhibition. Hence, we focused on negative correlation analysis between differently expressed miRNA and genes associated with metabolic, MAPK, cell adhesion and immune response pathways in LLC1 cells grown under 2D and 3D cell culture conditions. Indeed, we found that differently expressed genes associated to these pathways could be potentially regulated by miRNAs differently expressed in LLC1 cells.

In the present study the miRNA target filter analysis identified miRNAs showing inverse correlations with metabolic genes indicating the role of miRNA in metabolic pathway regulation. For instance, the down-regulation of miR-9, miR-19a, miR-135a, miR-495 and miR-590 negatively correlated with the up-regulation of genes involved in polysaccharide synthesis including alpha-mannosidase *man2a1*, glycosyltransferase *ext1* and beta-1,3-galactosyltransferase *B3galt6*. We also found that the down-regulation of genes involved lipid metabolism including *pla2g2c*, *dhrs9*, *ppt1* and *sc5d* inversely correlated with the up-regulation of miR-297a, miR-346, miR-466b, miR-466d, miR-468 and miR-669b. In addition, our results also revealed that the differential expression of diacylglycerol kinase *dgkb* and inositol polyphosphate 5-phosphatase *ocrl* regulating lipid signaling and membrane trafficking inversely correlated with the expression of miR-9, miR-126, miR-590 and miR-466 g, respectively. These findings are supported by recent studies demonstrating important roles of miRNAs in metabolic rearrangement occurring in cancer cells [42, 43]. Furhtermore, our results indicated that the expression of *kras*, *mknk1* and *pak2* kinases involved in the MAP kinase pathway negatively correlated with the expression of miR-761, mir-195 and miR-297a, respectively. The target correlation analysis also depicted miR-34b, miR-34c, miR-466f and miR-500 miRNAs as potential negative regulators of *sos2* gene expression. However, the evidence implicating miRNAs role in MAP kinase pathway is still emerging. Previous report suggested that miR-34c may suppress proliferation of lung cancer cells by inhibition of MAPK pathway [44]. In addition, previous data also associated regulation of miRNAs with MAP kinases in pancreatic cancer cells showing that expression of miR-34a inversely correlated with MAPK pathway activity [45]. Ichimura et al. also demonstrated that miR-34a suppressed the expression of MEK1 leading to repression of the MEK-ERK signalling axis [46]. In the present study we also observed a significant link between deregulated expression of miRNA and cell adhesion molecules. For example, our results indicated a negative correlation between expression of *col1a1* and miR-135a, miR-137 and miR-590. In addition, decreased *flna* expression might be influenced by miR-328 and miR-761. These findings are consistent with a previous report indicating the presence of feedback mechanisms that promote ECM molecules, which are downstream targets of specific miRNA, to regulate expression of these miRNAs [40]. A similar target enrichment analysis also revealed that increased expression of miRNAs might be connected with the regulation of immune response pathway genes. For example, our results depicted a negative correlation between expression of chemokine receptor *xcr1* and miR-669b. Additionally, we also noted that decreased expression of *oas3* might be affected by numerous miRNAs. Thus, taken together these findings suggest that metabolic, MAP kinase, cell adhesion and immune response pathway genes might be regulated by miRNAs altered in ECM dependent manner. Therefore, the 3D cell culture model could be applied not only for further investigation of common cancer pathways altered in ECM dependent manner but also for the study of specific miRNAs involved in ECM-mediated cancer signaling networks. Further understanding of complex ECM dependent signaling networks in tumors could direct to novel cancer treatment strategies.

## Conclusions

In conclusion, our study identified significant changes in gene and miRNA expression that occurred in mouse Lewis lung carcinoma LLC1 cells during the shift to lr-ECM 3D cell culture conditions. Our findings suggest that 3D cell culture should be considered as a critical experimental approach to uncover the molecular regulation of genes and miRNA involved in tumor cell - tumor microenvironment interactions in vivo. Furthermore, results obtained under 3D cell culture conditions could also be strongly considered in preclinical targeted therapy development and hold the prognostic and therapeutic potential.

## Additional files

Additional file 1: Table S1. List of primer sequences. (PDF 68 kb)
Additional file 2: Table S2. Differentially expressed genes (fold change above 1.5; *P* < 0.05) in LLC1 cells grown under lr-ECM 3D versus 2D cell culture conditions. (XLS 2578 kb)
Additional file 3: Table S3. List of all KEGG Pathways enriched in differently expressed genes in LLC1 cells under lr-ECM 3D versus 2D cell culture conditions. (XLS 28 kb)
Additional file 4: Table S4. Full list of differentially expressed genes cells associated to metabolic pathways, MAP kinase, cell adhesion and immune response functional categories in LLC cells cultured in lr-ECM 3D versus 2D. (DOC 58 kb)
Additional file 5: TAble S5. Differentially expressed miRNA (fold change above 2; *P* < 0.05) in LLC1 cells grown under lr-ECM 3D versus 2D cell culture conditions. (XLS 45 kb)
Additional file 6: Table S6. Target analysis of differently expressed miRNAs in LLC1 cells under lr-ECM 3D versus 2D cell culture conditions. (XLS 1257 kb)
Additional file 7: Table S7. List of all KEGG Pathways enriched in differently expressed miRNAs in LLC1 cells grown under lr-ECM 3D versus 2D cell culture conditions. (XLS 32 kb)
Additional file 8: Hierarchical clustering analysis of upregulated (A) and downregulated (B) miRNA associated KEGG pathways. (PDF 508 kb)

## Abbreviations

2D: Two-dimensional
3D: Three-dimensional
ECM: Extracellular matrix
lr: Laminin rich
MAPK: Mitogen activated protein kinase
