# Supplementary material for: Gene and miRNA expression signature of Lewis lung carcinoma LLC1 cells in extracellular matrix enriched microenvironment
Source: BMC Cancer. 2016 Oct 11;16:789. doi: 10.1186/s12885-016-2825-9 (PMC5057255; doi:10.1186/s12885-016-2825-9)
Supplement: Additional file 1: Table S1. — List of primer sequences. (PDF 68 kb) [file 12885_2016_2825_MOESM1_ESM.pdf]

Additional file 1. List of primer sequences.

List of primer sequences used for gene qPCR

Gene	Forward sequence	Reverse sequence
Hnf4a	CACGCGGAGGTCAAGCTAC	CCCAGAGATGGGAGAGGTGAT
Ifnb1	CCGAGCAGAGATCTTCAGGAA	CCTGCAACCACCACTCATTCT
Klf8	TCAGAAAGTGGTTCGATGCAG	AACAGAGCTGGGTTCTCCATT
Fgfr4	GCTCGGAGGTAGAGGTCTTGT	CCACGCTGACTGGTAGGAA
Hprt1	CCTAAGATGAGCGCAAGTTGAA	CCACAGGACTAGAACACCTGCTAA

List of primer sequences used for miRNA cDNA synthesis

miRNAs	Stem-loop sequence
Mir-207	GTCGTATCCAGTGCAGGGTCCGAGGTATTCGCACTGGATACGACGAGGGA
miR-376c-3p	GTCGTATCCAGTGCAGGGTCCGAGGTATTCGCACTGGATACGACACGTGA
miR-466f-3p	GTCGTATCCAGTGCAGGGTCCGAGGTATTCGCACTGGATACGACGTGTGT
miR-195a-5p	GTCGTATCCAGTGCAGGGTCCGAGGTATTCGCACTGGATACGACGCCAAT
Sno135	GTCGTATCCAGTGCAGGGTCCGAGGTATTCGCACTGGATACGACCTTCAG

List of primer sequences used for miRNA qPCR

miRNAs	Forward sequence	Reverse sequence
Mir-207	CGGCTTCTCCTGGCTCTCC	CAGTGCAGGGTCCGAGGT
miR-376c-3p	CGGCGAACATAGAGGAAATT	CAGTGCAGGGTCCGAGGT
miR-466f-3p	GGCGCATACACACACACAT	CAGTGCAGGGTCCGAGGT
miR-195a-5p	GGCGTAGGTAGTTTCATGTT	CAGTGCAGGGTCCGAGGT
Sno135	CGGTGGTGAGCCTATGGTTTT	CAGTGCAGGGTCCGAGGT
