# Supplementary material for: Gene and miRNA expression signature of Lewis lung carcinoma LLC1 cells in extracellular matrix enriched microenvironment
Source: BMC Cancer. 2016 Oct 11;16:789. doi: 10.1186/s12885-016-2825-9 (PMC5057255; doi:10.1186/s12885-016-2825-9)
Supplement: Additional file 4: Table S4. — Full list of differentially expressed genes cells associated to metabolic pathways, MAP kinase, cell adhesion and immune response functional categories in LLC cells cultured in lr-ECM 3D versus 2D. (DOC 58 kb) [file 12885_2016_2825_MOESM4_ESM.doc]

**Supplementary table 4.** Full list of differentially expressed genes cells associated to metabolic pathways, MAP kinase, cell adhesion and immune response functional categories in LLC cells cultured in lr-ECM 3D versus 2D.

| **Category** | **ALL** | | |  | **Up-regulated** | | | | **Down-regulated** | | |
| --- | --- | --- | --- | --- | --- | --- | --- | --- | --- | --- | --- |
|  |  | Genes | p value |  | Genes | | p value |  | Genes | | p value |
| **Metabolic pathways** | 73 | Ggt1, Cyp2j11, Ido2, Lclat1, Dtymk, Pcyt1b, Pmvk, Pfkm, Pfkp, Cds1, Adssl1, Ckmt2, B4galt2, Kynu, Pigx, Pla2g2c, Aco2, Ppt1, Cda, Atp6v0c, Otc, Akr1b7, Gcnt2,  Gfpt1, Tdo2, Dgkb, Fut1, B3galt6,  Sphk1, Dhrs9, Nt5c, Mthfr, Man2a1  Ext1, Mat2b, Aldh1a2, St8sia5, Aanat, Aox1, Nat3, Ctps, Sc5d, B3gnt5, B3gat1, Itpa, Pigm, Lta4h,  Polr3g, Sdha, P4ha1, Pla2g3, Dgkq,  Polr3k, Paics, Xdh, Cad, Hpd, Cbr2,  Agpat3, Pole, Atp6v0e, Acsl5, Ocrl  Cyp4f18, Alg5, Pla2g12a, Tkt, Hyal6, Polr2j, Hsd17b3, Hadha,  Papss1, Ggt7 | 2.83e-13 |  | 30 | Nt5c, Ggt1,  Cyp2j11, Dgkq, Man2a1, Mat2b, Pla2g3, Ext1, Atp6v0c, Dtymk, Cad, Xdh, Pfkm, Hpd, Akr1b7,  Pfkp, Aox1, Agpat3, Ctps,  Cds1, Dgkb,  Adssl1, Cyp4f18,  Pla2g12a, B4galt2, Tkt, Polr2j, Hsd17b3,  B3galt6, Pigm | 7.55e-06 |  | 43 | Ido2, Mthfr,  Lclat1, ldh1a2,  Pcyt1b, Pmvk,  St8sia5, Aanat,  Nat3, Sc5d,  B3gnt5, Ckmt2,  B3gat1, Itpa,  Kynu, Pigx,  Pla2g2c, olr3g,  Lta4h, Aco2,  Sdha, P4ha1,  Ppt1, Cda, Polr3k, Paics, Otc, Gcnt2,  Cbr2, Tdo2, Gfpt1, tp6v0e,  Pole, Acsl5,  Ocrl, Fut1, Alg5, Hyal6, Hadha, Sphk1, Dhrs9, Papss1, Ggt7 | 2.02e-08 |
| **MAPK signaling pathway** | 25 | Elk4, Fgfr4, Cacng5, Pla2g3, Ppp3cc, Fgf20, Nfatc4, Ecsit, Cacna1d, Kras, Mapk8ip3, Rasgrp4, Pak2, Gng12, Flnc, Stmn1, Map3k14, Traf6, Sos2, Mknk1, Flna, Fgfr3, Pla2g12a, Pla2g2c, Ikbkg | 6.23e-08 |  | 11 | Elk4, Fgfr4, Pla2g3, Traf6, Ppp3cc, Pla2g12a, Cacna1d, Nfatc4, Mapk8ip3, Ikbkg, Gng12 | 0.0010 |  | 14 | Cacng5, Flnc, Stmn1, Map3k14, Mknk1, Sos2, Fgf20, Flna, Fgfr3, Ecsit, Kras, Rasgrp4, Pla2g2c, Pak2 | 0.0002 |
| **Regulation of actin cytoskeleton** | 20 | Fgfr4, Diap3, Fgf20, Pik3r2, Pak3, Kras, Itgb7, Pak2, Ppp1cb, Gng12, Myh10, Slc9a1, Sos2, Arhgef4, Ssh1, Fgfr3, Tmsb4x, RhoaItgal  Ssh3 | 1.35e-06 |  | 6 | Myh10, Tmsb4x, Fgfr4, Slc9a1, Arhgef4, Gng12 | 0.0404 |  | 14 | Diap3, Sos2, Fgf20, Ssh1, Fgfr3, Pik3r2, Pak3, Kras, Rhoa, Itgal, Itgb7, Pak2, Ssh3, Ppp1cb | 3.76e-05 |
| **Focal adhesion** | 17 | Lamb3, Vasp, Pdpk1, Pik3r2, Pak3, Vegfa, Itgb7, Pak2, Ppp1cb, Chad, Flnc, Sos2, Flna, Col3a1, Rhoa, Col1a1, Cav3 | 3.33e-05 |  | 7 | Lamb3, Pdpk1, Vegfa, Col1a1, Chad, Cav3, Col3a1 | 0.0148 |  | 10 | Vasp, Flnc, Sos2, Flna, Pik3r2, Pak3, Rhoa, Itgb7, Pak2, Ppp1cb | 0.0018 |
| **Cell adhesion molecules (CAMs)** | 10 | Cldn16, 4930412D23Rik, Cdh4, H2-Bl, Cadm1, Nrcam, Itgal, Itgb7, Cd8a, Cntn2 | 0.0062 |  | 5 | Cadm1, Cldn16, Nrcam, Cdh4, Cntn2 | 0.0368 |  | 5 | 4930412D23Rik, Itgal, Itgb7, Cd8a, H2-Bl | NS |
| **Gap junction** | 7 | Grm1, Tubb2a, Gnas, Kras, Htr2c, Sos2, Itpr1 | 0.0103 |  | 2 | Tubb2a, Itpr1 | NS |  | 5 | Grm1, Gnas, Kras, Htr2c, Sos2 | 0.0174 |
| **Tight junction** | 9 | Ppp2r1b, Myh10, Cldn16, 4930412D23Rik, Pard3, Ppp2r2b, Kras, Rhoa, Inadl | 0.0103 |  | 5 | Cldn16, Inadl, Myh10, Pard3, Ppp2r2b | NS |  | 4 | 4930412D23Rik, Kras, Rhoa, Ppp2r1b | NS |
| **ECM-receptor interaction** | 6 | Lamb3, Itgb7, Col1a1, Chad, Col3a1, Agrn | 0.0237 |  | 4 | Lamb3, Col1a1, Chad, Col3a1 | 0.0257 |  | 2 | Itgb7, Agrn | NS |
| **Cytokine-cytokine receptor interaction** | 18 | Ltb, Xcr1, Il12rb2, Tnfrsf11a, Cxcr7, Ifnb1, Acvr2a, Vegfa, Tnfrsf1b, Epo, Tnfrsf9, Il22ra1, Il21r, Tnfrsf25, Ccr3, Bmpr1a, Cntfr, Il2ra | 0.0001 |  | 6 | Ifnb1, Il21r, Il22ra1, Ltb, Tnfrsf9, Vegfa | NS |  | 12 | Tnfrsf25, Ccr3, Xcr1, Bmpr1a, Tnfrsf11a, Il12rb2, Cxcr7, Cntfr, Acvr2a, Il2ra, Tnfrsf1b, Epo | 0.0011 |
| **T cell receptor signaling pathway** | 11 | Map3k14, Sos2, Ppp3cc, Pik3r2, Pak3, Nfatc4, Kras, Rhoa, Ikbkg, Cd8a, Pak2 | 0.0003 |  | 3 | Ikbkg, Nfatc4,  Ppp3cc | NS |  | 8 | Map3k14, Sos2, Pik3r2, Pak3, Kras, Rhoa, Cd8a, Pak2 | 0.0011 |
| **VEGF signaling pathway** | 9 | Nfatc4, Pla2g3, Kras, Vegfa, Sphk1, Pla2g2c, Ppp3cc, Pla2g12a, Pik3r2 | 0.0004 |  | 5 | Nfatc4, Pla2g3, Vegfa, Ppp3cc, Pla2g12a | 0.0062 |  | 4 | Kras, Sphk1, Pla2g2c, Pik3r2 | NS |
| **Cytosolic DNA-sensing pathway** | 7 | Polr3k, Tbk1, Ifnb1, Ddx58, Irf3, Ikbkg, Polr3g | 0.0013 |  | 4 | Tbk1, Ifnb1, Ddx58, Ikbkg | 0.0111 |  | 3 | Polr3k, Irf3, Polr3g | NS |
| **B cell receptor signaling pathway** | 7 | Nfatc4, Lilrb3, Kras, Sos2, Ppp3cc, Ikbkg, Pik3r2 | 0.0058 |  | 3 | Nfatc4, Ppp3cc, Ikbkg | NS |  | 4 | Lilrb3, Kras, Sos2, Pik3r2 | NS |
| **RIG-I-like receptor signaling pathway** | 6 | Tbk1, Ifnb1, Ddx58, Irf3, Traf6, Ikbkg | 0.0121 |  | 5 | Tbk1, Ifnb1, Ddx58, Traf6, Ikbkg | 0.0045 |  | 1 | Irf3 | NS |
| **Natural killer cell mediated cytotoxicity** | 8 | Klra7, Sos2, Ppp3cc, Pik3r2, Nfatc4, Ifnb1, Kras, Itgal | 0.0153 |  | 3 | Ppp3cc, Nfatc4, Ifnb1 | NS |  | 5 | Kras, Klra7, Itgal, Sos2, Pik3r2 | 0.0435 |
| **Jak-STAT signaling pathway** | 9 | Cntfr, Ifnb1, Il2ra, Sos2, Epo, Il12rb2, Il21r, Pik3r2, Il22ra1 | 0.0159 |  | 3 | Ifnb1, Il21r, Il22ra1 | NS |  | 6 | Cntfr, Il2ra, Sos2, Epo, Il12rb2, Pik3r2 | 0.0336 |
| **Fc epsilon RI signaling pathway** | 6 | Pla2g3, Kras, Pla2g2c, Sos2, Pla2g12a, Pik3r2 | 0.0186 |  | 2 | Pla2g3, Pla2g12a | NS |  | 4 | Kras Pla2g2c, Sos2, Pik3r2 | NS |
| **Chemokine signaling pathway** | 9 | Ccr3, Xcr1, Sos2, Pik3r2, Pard3, Kras, Rhoa, Ikbkg, Gng12 | 0.0362 |  | 3 | Gng12, Ikbkg,  Pard3 | NS |  | 6 | Ccr3, Kras, Pik3r2, Rhoa,  Sos2, Xcr1 | NS |
| **Toll-like receptor signaling pathway** | 5 | Tbk1, Ifnb1, Ifnb1, Traf6, Ikbkg, Pik3r2 | 0.0401 |  | 4 | Tbk1, Ifnb1, Traf6, Ikbkg | NS |  | 1 | Pik3r2 | NS |
