# Supplementary material for: Gene and miRNA expression signature of Lewis lung carcinoma LLC1 cells in extracellular matrix enriched microenvironment
Source: BMC Cancer. 2016 Oct 11;16:789. doi: 10.1186/s12885-016-2825-9 (PMC5057255; doi:10.1186/s12885-016-2825-9)
Supplement: Additional file 8: — Hierarchical clustering analysis of upregulated (A) and downregulated (B) miRNA associated KEGG pathways. (PDF 508 kb) [file 12885_2016_2825_MOESM8_ESM.pdf]

Additional file 8. Hierarchical clustering analysis of upregulated (A) and downregulated (B) miRNA associated KEGG pathways.

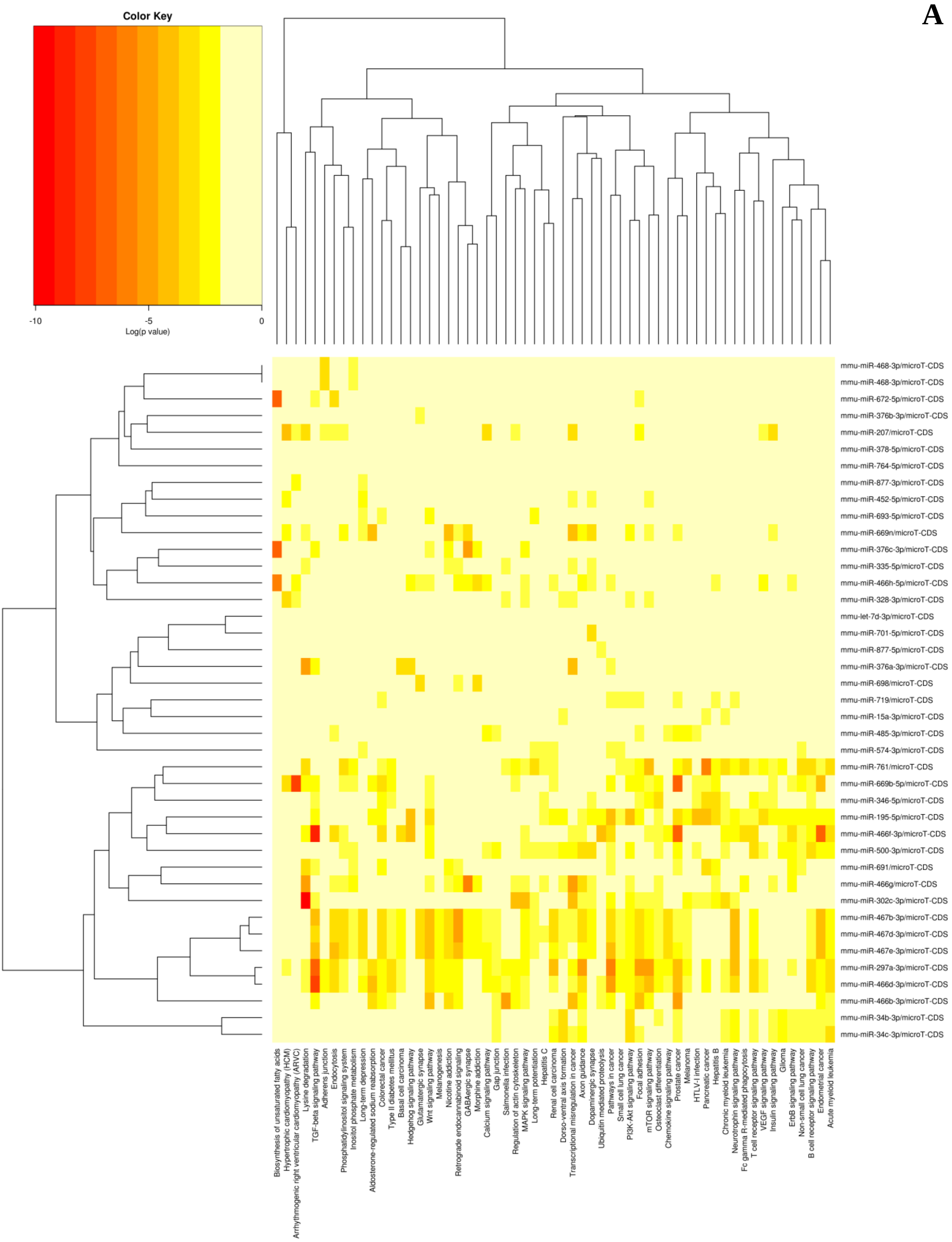

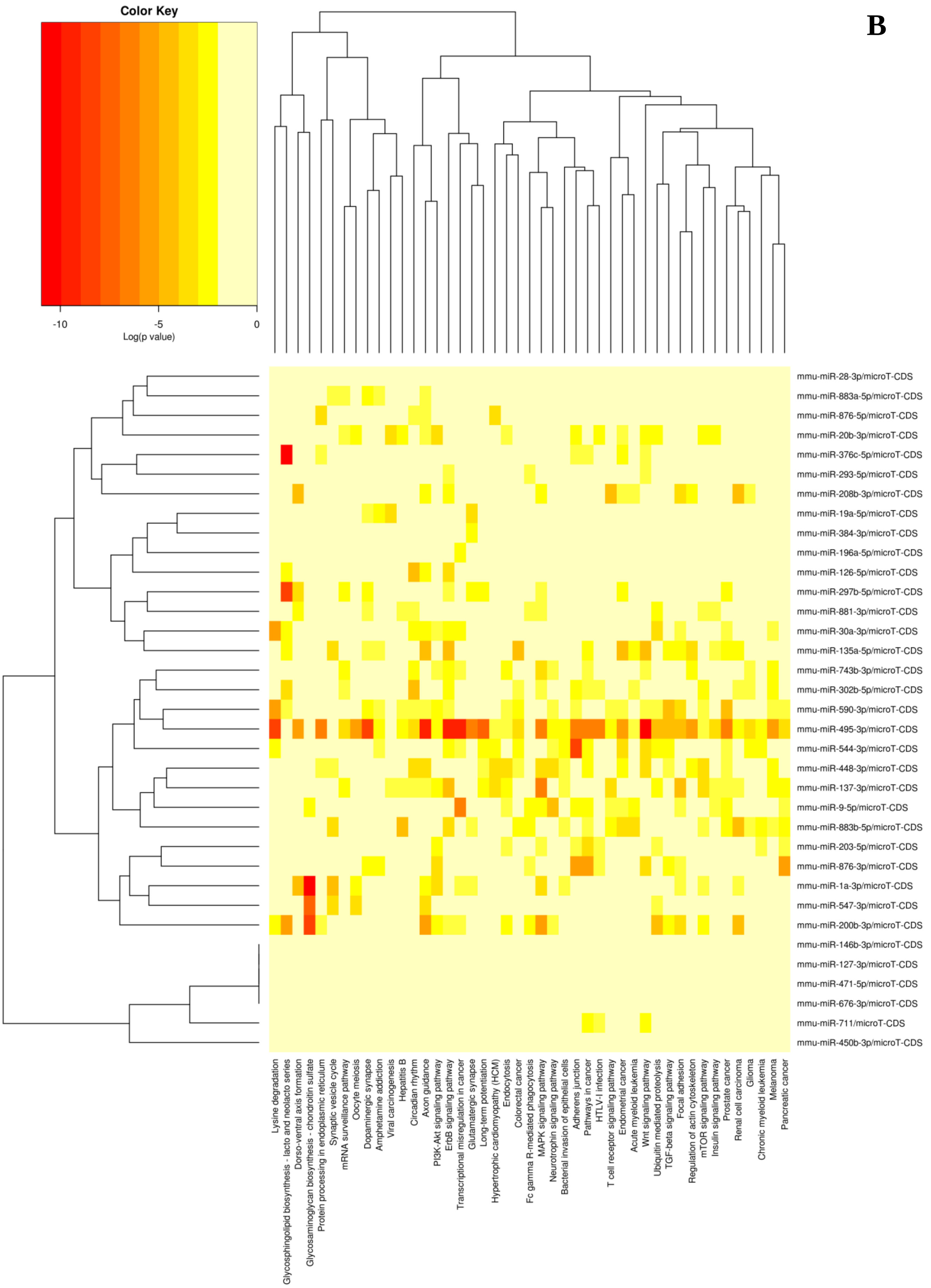

B
Color Key
-10
-5
0
Log(p value)
mmu-miR-28-3p/microT-CDS
mmu-miR-883a-5p/microT-CDS
mmu-miR-876-5p/microT-CDS
mmu-miR-20b-3p/microT-CDS
mmu-miR-376c-5p/microT-CDS
mmu-miR-293-5p/microT-CDS
mmu-miR-208b-3p/microT-CDS
mmu-miR-19a-5p/microT-CDS
mmu-miR-384-3p/microT-CDS
mmu-miR-196a-5p/microT-CDS
mmu-miR-126-5p/microT-CDS
mmu-miR-297b-5p/microT-CDS
mmu-miR-881-3p/microT-CDS
mmu-miR-30a-3p/microT-CDS
mmu-miR-135a-5p/microT-CDS
mmu-miR-743b-3p/microT-CDS
mmu-miR-302b-5p/microT-CDS
mmu-miR-590-3p/microT-CDS
mmu-miR-495-3p/microT-CDS
mmu-miR-544-3p/microT-CDS
mmu-miR-448-3p/microT-CDS
mmu-miR-137-3p/microT-CDS
mmu-miR-9-5p/microT-CDS
mmu-miR-883b-5p/microT-CDS
mmu-miR-203-5p/microT-CDS
mmu-miR-876-3p/microT-CDS
mmu-miR-1a-3p/microT-CDS
mmu-miR-547-3p/microT-CDS
mmu-miR-200b-3p/microT-CDS
mmu-miR-146b-3p/microT-CDS
mmu-miR-127-3p/microT-CDS
mmu-miR-471-5p/microT-CDS
mmu-miR-676-3p/microT-CDS
mmu-miR-711/microT-CDS
mmu-miR-450b-3p/microT-CDS
Lysine degradation
Glycosphingolipid biosynthesis - lacto and neolacto series
Dorso-ventral axis formation
Glycosaminoglycan biosynthesis - chondroitin sulfate
Protein processing in endoplasmic reticulum
Synaptic vesicle cycle
mRNA surveillance pathway
Oocyte meiosis
Dopaminergic synapse
Amphetamine addiction
Viral carcinogenesis
Hepatitis B
Circadian rhythm
Axon guidance
PI3K-Akt signaling pathway
ErbB signaling pathway
Transcriptional misregulation in cancer
Glutamatergic synapse
Long-term potentiation
Hypertrophic cardiomyopathy (HCM)
Endocytosis
Colorectal cancer
Fc gamma R-mediated phagocytosis
MAPK signaling pathway
Neurotrophin signaling pathway
Bacterial invasion of epithelial cells
Adherens junction
Pathways in cancer
HTLV-I infection
T cell receptor signaling pathway
Endometrial cancer
Acute myeloid leukemia
Wnt signaling pathway
Ubiquitin mediated proteolysis
TGF-beta signaling pathway
Focal adhesion
Regulation of actin cytoskeleton
mTOR signaling pathway
Insulin signaling pathway
Prostate cancer
Renal cell carcinoma
Glioma
Chronic myeloid leukemia
Melanoma
Pancreatic cancer
